# OK432 sclerotherapy for refractory chylous leakage after neck dissection

**DOI:** 10.1093/jscr/rjad310

**Published:** 2023-06-15

**Authors:** Yuiko Hashimoto, Masanori Teshima, Mitsuko Yui, Shun Tatehara, Keisuke Iritani, Tatsuya Furukawa, Hirotaka Shinomiya, Ken-ichi Nibu

**Affiliations:** Department of Otolaryngology-Head and Neck Surgery, Kobe University Graduate School of Medicine, 7-5-1, Kusunoki-Cho, Chuo-Ku, Kobe, Hyogo, 650-0017, Japan; Department of Otolaryngology-Head and Neck Surgery, Kobe University Graduate School of Medicine, 7-5-1, Kusunoki-Cho, Chuo-Ku, Kobe, Hyogo, 650-0017, Japan; Department of Otolaryngology-Head and Neck Surgery, Kobe University Graduate School of Medicine, 7-5-1, Kusunoki-Cho, Chuo-Ku, Kobe, Hyogo, 650-0017, Japan; Department of Otolaryngology-Head and Neck Surgery, Kobe University Graduate School of Medicine, 7-5-1, Kusunoki-Cho, Chuo-Ku, Kobe, Hyogo, 650-0017, Japan; Department of Otolaryngology-Head and Neck Surgery, Kobe University Graduate School of Medicine, 7-5-1, Kusunoki-Cho, Chuo-Ku, Kobe, Hyogo, 650-0017, Japan; Department of Otolaryngology-Head and Neck Surgery, Kobe University Graduate School of Medicine, 7-5-1, Kusunoki-Cho, Chuo-Ku, Kobe, Hyogo, 650-0017, Japan; Department of Otolaryngology-Head and Neck Surgery, Kobe University Graduate School of Medicine, 7-5-1, Kusunoki-Cho, Chuo-Ku, Kobe, Hyogo, 650-0017, Japan; Department of Otolaryngology-Head and Neck Surgery, Kobe University Graduate School of Medicine, 7-5-1, Kusunoki-Cho, Chuo-Ku, Kobe, Hyogo, 650-0017, Japan

**Keywords:** head and neck cancer, neck dissection, chylous leakage, intractable, complication

## Abstract

Chylous leakage is a rare but serious postoperative complication of neck dissection (ND). Most chylous leakages are successfully treated either by drainage or ligation of the thoracic duct, but the resolution is occasionally prolonged. OK432 sclerotherapy is used to treat various refractory cystic diseases of the head and neck. Three patients were treated with OK432 sclerotherapy for refractory chylous leakage following ND. Case 1 involved a 77-year-old man with chylous leakage after a total laryngectomy and bilateral ND. Case 2 involved a 71-year-old woman who underwent total thyroidectomy and left ND for thyroid cancer. Case 3 involved a 61-year-old woman who underwent right ND for oropharyngeal cancer. In all patients, chylous leakage rapidly improved after OK432 injection without any complications. Our results suggest the efficacy of OK432 sclerotherapy in patients with refractory chylous leakage after ND.

## INTRODUCTION

Chylous leakage is a relatively rare postoperative complication of neck dissection (ND), with an incidence of 1–2% [1]. Chylous leakage occurs due to an inadvertent injury to the thoracic duct or its branches in the lower neck. Despite its low incidence, it can be a life-threatening postoperative complication due to the increased risk of infection, bleeding, hypovolemia, electrolyte imbalance, and malnutrition and may cause delayed wound healing [2]. In most cases, chylous leakage is improved by fasting or administration of a fat-restricted diet, wound compression, drainage, or ligation of the thoracic duct or its branches. However, the resolution is occasionally prolonged and can be potentially life-threatening.

OK432 (CHUGAI Pharmaceutical Co. LTD., Tokyo, Japan) is a lyophilised streptococcal preparation prepared from a strain of the A-group *Streptococcus pyogenes* incubated with penicillin. Although OK432 was originally developed as an immunopotentiator for cancer, it is now widely accepted as a safe and effective material for sclerotherapy of pleural effusion and various cystic lesions in the head and neck [3]. Encouraged by these reports, we successfully treated three patients with refractory cervical chylous leakage with OK432 sclerotherapy. Herein, we report the details of the procedure and results with a literature review.

## CASE REPORTS

### Case 1

**Figure 1:**
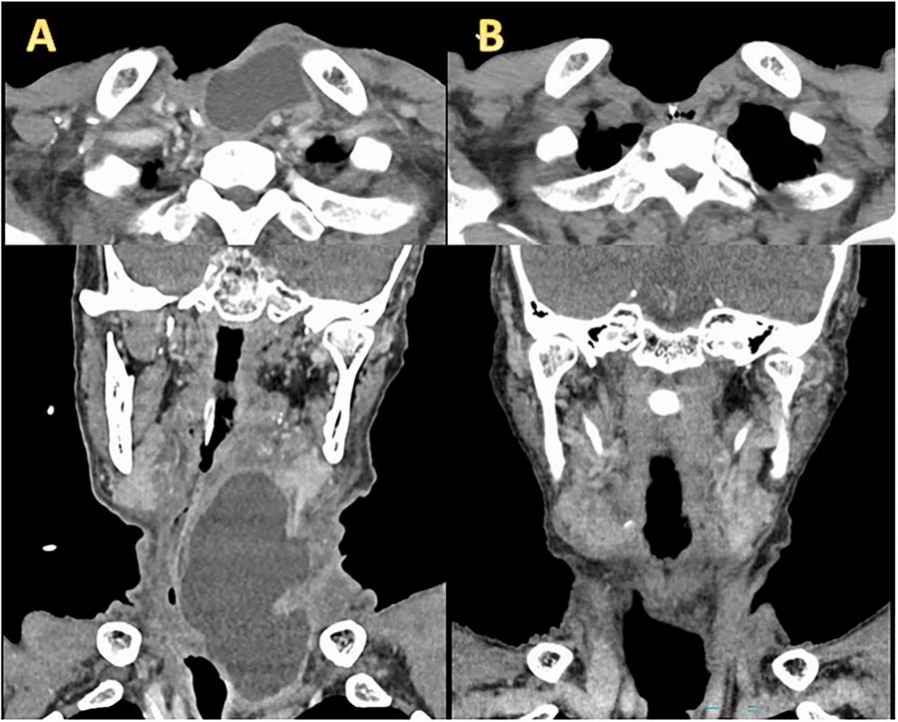
Enhanced CT before and after OK432 injection. (**A**) Before OK432 injection. Cervical CT shows a chylous leakage in the left supraclavicular fossa. (**B**) After two cycles of OK4432 injection. The chylous leakage completely disappeared after OK432 sclerotherapy.

A 77-year-old man underwent a total laryngectomy with modified bilateral ND for advanced laryngeal squamous cell carcinoma (cT4aN0M0, stage IVA). A small chylous leakage was observed on postoperative day (POD) 1. As the chylous leakage did not improve with fasting, wound compression, and drainage, ligation of the thoracic duct and its branches was attempted under general anesthesia on POD3. However, the leakage persisted, and computed tomography (CT) on POD17 revealed a lymphatic cyst extending from the peritonsillar space to the upper mediastinum (Fig. 1A). Therefore, we decided to administer OK432 sclerotherapy. After draining the leaked chyle, we injected 2KE of OK432 diluted with 4 mL of normal saline and compressed the wound. The day after the injection, the chylous leakage completely stopped without complications. Seven days after the first injection, a second dose of OK432 was administered in a similar manner. CT on POD 83 confirmed the complete disappearance of the retained lymph (Fig. 1B).

### Case 2

A 71 year-old female underwent total thyroidectomy with bilateral ND for papillary thyroid carcinoma (cT4aN1bM0, Stage III). The postoperative course was uneventful, but fluid retention was palpable in the lower part of the left anterior neck on POD2. As the chylous leakage did not improve with fasting, drainage, and compression, ligation of the thoracic duct and its branches was attempted under general anesthesia on POD4. Although the chylous leak stopped after ligation, it recurred on POD7. Therefore, we decided to administer OK432 sclerotherapy. After aspirating as much retained lymph as possible using a syringe, 3KE of OK432 diluted in 6 mL of normal saline was injected using an 18G needle from the left supraclavicular fossa. Chylous leakage completely stopped immediately after the injection without any complications.

### Case 3

**Figure 2:**
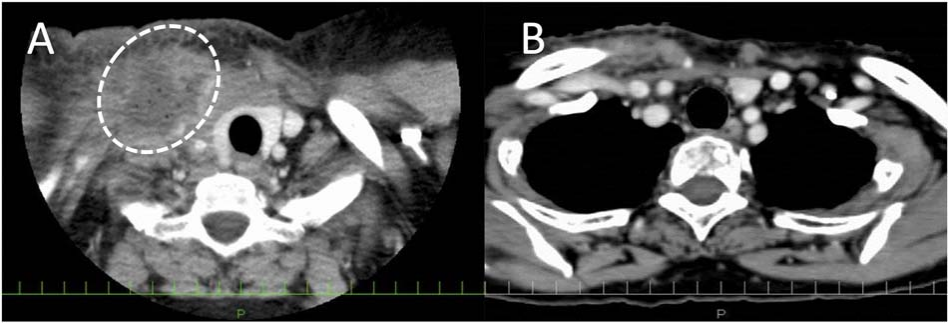
Enhanced CT before and after OK432 injection. (**A**) Before OK432 injection. The lymph (dashed circle) is retained in the right supraclavicular fossa. (**B**) After injection. The lymphatic leakage disappeared after OK432 sclerotherapy.

A 61-year-old female underwent right ND for recurrent oropharyngeal squamous cell carcinoma (rT0N2bM0). The postoperative course was uneventful, but the milky fluid was noted in the suction reservoir on POD1 (Fig. 2A). As the chyle leakage did not improve with administration of a fat-restricted diet, wound compression, and drainage, after aspirating as much retained lymph as possible using a syringe, 3KE of OK432 diluted in 6 mL of normal saline was injected from the right supraclavicular fossa on POD16. After OK432 injection, chylous leakage immediately stopped without any adverse events, such as fever and local pain (Fig. 2B).

## DISCUSSION

Postoperative chylous leakage after ND is relatively rare [1, 4, 5]. However, once chylous leakage occurs, it takes time to improve. Moreover, the patient’s condition deteriorates, wound healing is delayed due to hypoalbuminemia, and the risk of wound infection increases. Conservative treatment including fasting, administration of a fat-restricted diet, drainage, and wound compression and surgical treatment such as drainage and ligation of the thoracic duct and its branches are routinely performed for the treatment of chylous leakage [6]. In addition to these, recently, several authors reported the usefulness of octreotide [7] and ultrasound-guided intranodal lymphangiography [8, 9]. However, occasionally, none of these works.

OK432 was originally developed as an immunopotentiator for malignant neoplasms. OK432 triggers severe inflammation at the injection site and promotes the release of various cytokines, which are believed to potentiate immunoactivity against cancer [10]. Recently, a few studies suggested that OK432 is an easy, safe, and effective treatment for ranula extending to the parapharyngeal space and thyroglossal duct cysts [11, 12] that can be used as a first-line treatment for ranula extending to the parapharyngeal space. Currently, OK432 is widely used to reduce ascites and pleural effusion. When injected into the peritoneal or pleural cavity, OK432 causes inflammation. Consequently, the cavity collapses owing to the adhesion of the lining membrane.

Encouraged by these applications of OK432, we treated refractory chylous leakage using OK432 sclerotherapy for the first time. Chylous leakage stopped immediately after OK432 injection, without any adverse events. The risk of chylous leakage inevitably increases when multiple metastatic lymph nodes are present in the lateral neck [5]. The present series shows that chylous leakage develops despite meticulous care during surgical procedures around the thoracic duct near the lower part of the internal jugular vein. In addition, once chylous leakage develops, identification of the thoracic duct in the surgical field is difficult even under general anesthesia, and ligation often fails, as in cases 1 and 2. Based on our experience with the first two cases, we treated case 3 with OK432 sclerotherapy without attempting ligation. Previous studies have reported the possibility of brachial plexus injury during posterior cervical dissection for surgical repair of a mastoid fistula. In contrast, OK432 sclerotherapy is safe [13]. Case 1 had liver cirrhosis. In cirrhotic patients, the thoracic duct diameter is reported to be two to four times greater with a 3–6-fold increase in ductal flow rate, which causes massive chylous leakage [14]. Although many studies recommend surgical management using octreotide over conservative treatment for chyle leakage in patients with cirrhosis, sclerotherapy was effective in this patient.

Although no adverse effects were observed in any patient in the present series, infusion reactions such as local pain, inflammation, edema, and fever should be considered as possible complications of OK432 injection, based on the experience with other cystic lesions in the head and neck. Our current strategy for patients with chylous leakage after ND is to open the wound and insert a Penrose drain. If the chyle leakage does not stop after several days of compression, we inject OK432, remove the Penrose drain, and apply compression. In all cases, the chylous leakage stopped, and the patient recovered without any adverse events.

## CONCLUSION

Herein, we reported three cases of refractory chylous leakage after ND that were successfully treated with OK432 sclerotherapy without any complications. Although the number of patients was small, we recommend OK432 sclerotherapy as a safe and effective treatment option for patients with refractory chylous leakage after ND.

## CONFLICT OF INTEREST STATEMENT

None declared.

## FUNDING

None.

## ETHICS APPROVAL

The Ethics Committee confirms that ethics approval for case reports or case series is waived.
